# Enhanced Transfection of Human Mesenchymal Stem Cells Using a Hyaluronic Acid/Calcium Phosphate Hybrid Gene Delivery System

**DOI:** 10.3390/polym11050798

**Published:** 2019-05-04

**Authors:** Jung Eun Lee, Yue Yin, Su Yeon Lim, E. Seul Kim, Jaeback Jung, Dahwun Kim, Ji Won Park, Min Sang Lee, Ji Hoon Jeong

**Affiliations:** School of Pharmacy, Sungkyunkwan University, Suwon 16419, Korea; jelee1213@naver.com (J.E.L.); yinyue2013@skku.edu (Y.Y.); suuyeon37@skku.edu (S.Y.L.); seul4146@naver.com (E.S.K.); domonde@naver.com (J.J.); gnjs0219@naver.com (D.K.); jwjwpar@gmail.com (J.W.P.)

**Keywords:** human mesenchymal stem cell, calcium phosphate nanoparticle, catechol modified, hyaluronic acid, gene delivery, osteogenic differentiation

## Abstract

Human mesenchymal stem cells (hMSCs) show enormous potential in regenerative medicine and tissue engineering. However, current use of hMSCs in clinics is still limited because there is no appropriate way to control their behavior in vivo, such as differentiation to a desired cell type. Genetic modification may provide an opportunity to control the cells in an active manner. One of the major hurdles for genetic manipulation of hMSCs is the lack of an efficient and safe gene delivery system. Herein, biocompatible calcium phosphate (CaP)-based nanoparticles stabilized with a catechol-derivatized hyaluronic acid (dopa-HA) conjugate were used as a carrier for gene transfection to hMSCs for improved differentiation. Owing to the specific interactions between HA and CD44 of bone marrow-derived hMSCs, dopa-HA/CaP showed significantly higher transfection in hMSCs than branched polyethylenimine (bPEI, MW 25 kDa) with no cytotoxicity. The co-delivery of a plasmid DNA encoding bone morphogenetic protein 2 (BMP-2 pDNA) and micro RNA 148b (miRNA-148b) by dopa-HA/CaP achieved significantly improved osteogenic differentiation of hMSCs.

## 1. Introduction

Mesenchymal stem cells (MSCs) have the potential to differentiate into osteoblasts, adipocytes, myocytes, chondrocytes, and neuronal cells [1]. The differentiation of each cell type is dependent on the activation of various cytokines and transcription factors. Several regulators, including cytokines, affect osteogenic differentiation in mesenchymal progenitor cells. Among them, bone morphogenetic proteins (BMPs) are reported to play important roles for embryogenesis [2], organogenesis [3,4], cell proliferation [5,6], and stem cell differentiation [7]. Particularly, BMP-2 provides an early-stage signal for osteogenic differentiation from mesenchymal progenitor cells [8,9,10,11]. It was originally identified by its ability to induce the formation of cartilage and bone [9,10,11,12]. A few antagonists have also been found to regulate BMP activities. Among these, Noggin plays an important role in the regulation of BMP-mediated differentiation by directly interacting with BMPs. Noggin and BMP interactions preclude BMPs from binding to their cell surface receptors and initiating signal transduction in cells. This suggests that Noggin directly regulates the rate of cell differentiation by interacting with BMPs. The suppression of Noggin using a specific miRNA increases the BMP-mediated osteogenic differentiation of hMSCs [13,14].

Micro-RNAs (miRNAs), non-coding RNAs consisting of 21–25 nucleotides, bind to complementary sequences in the 3’ untranslated region or coding region of target mRNAs and regulate the expression of those genes at a post-transcriptional level [15,16]. Recently, miRNAs have emerged as important regulators in tumorigenesis, viral infection, and cell differentiation [17]. Hence, various miRNAs have been used for differentiation of osteogenesis [18,19,20]. It was reported that miRNA-148b (mir148b) can be used to induce osteogenic differentiation by blocking the Noggin signal [16,21,22]. 

Calcium phosphate (CaP) has long been used as a non-viral gene carrier for in vitro transfection due to its excellent biocompatibility and low toxicity [23,24,25]. However, CaP-mediated gene delivery has low transfection efficiency and poor reproducibility due to rapid and uncontrollable growth and aggregation of CaP nanoparticles [26]. In our previous study, we showed that a conjugate of hyaluronic acid (HA) and 3,4-dihydroxy-L-phenylalanine (dopa) (dopa-HA), an unusual amino acid found in adherent threads of marine mussels, can effectively stabilize CaP nanoparticles containing pDNA and siRNA. Dopa has been widely investigated as a surface modification agent owing to its excellent adhesive properties to a variety of organic and inorganic materials, especially in wet conditions. In addition, a catechol group of dopa provides efficient binding to hydroxyapatite, a crystalline form of calcium phosphate [27,28]. In this study, we took advantage of a dopa-HA conjugate to control CaP growth and deliver desired genes to hMSCs through the specific interactions between HA and CD44, which are highly expressed in hMSCs [29,30]. The simultaneous delivery of mir148b and a plasmid DNA encoding BMP-2 (pBMP-2) using dopa-HA/CaP could improve osteogenic differentiation of hMSCs in a synergistic manner by blocking the Noggin signal and stimulating differentiation at the same time.

## 2. Materials and Methods 

### 2.1. Materials

Hyaluronic acid (HA, MW 50 kDa) was obtained from Lifecore Biomedical (Chaska, MN, USA). Calcium chloride (CaCl_2_), disodium hydrogen phosphate (Na_2_HPO_4_), acetic acid, branched polyethylenimine (bPEI, MW 25 kDa), dopamine hydrochloride, *N*-(3-dimethylaminopropyl)-*N*′-ethylcarbodiimide hydrochloride (EDC), *N*-hydroxysuccinimide (NHS), hydrochloric acid (HCl), dimethylsulfoxide (DMSO), 4′,6-diamidino-2-phenylindole (DAPI), and thiazolyl blue tetrazolium bromide (MTT) were bought from Sigma-Aldrich (St. Louis, MO, USA). BMP-2 cDNA ORF Clone was purchased from Sino Biological, Inc. (Wayne, PA, USA), and mir148b was acquired from Bioneer (Daejeon, Korea). Dulbecco’s modified Eagle’s Medium (DMEM) and fetal bovine serum (FBS) were obtained from HyClone (Logan, UT, USA). Ethidium monoazide (EMA) was purchased from Invitrogen (Carlsbad, CA, USA). The luciferase assay system kit was bought from Promega Corp. (Madison, WI, USA).

### 2.2. Synthesis of Dopamine-Conjugated HA (Dopa-HA)

The dopa-HA conjugate was prepared by coupling reaction between the carboxyl group of HA and an amine group of dopamine using EDC/NHS chemistry [31]. Briefly, HA (1 g, 20 μmol) was dissolved in 200 mL of degassed deionized water under nitrogen for 5 h. EDC (100.4 mg, 0.52 mmol) and NHS (60.7 mg, 0.52 mmol) were added to the HA solution. After 20 min, dopamine hydrochloride (99.4 mg, 0.52 mmol) was slowly added. The pH was adjusted to 5.5 using 0.1 M HCl, and the solution was reacted overnight at room temperature. Unreacted residues and byproducts were then removed with a dialysis membrane (MW cut off = 10 kDa) in distilled water (pH 5). The final products were lyophilized and stored at −20°C. The structure of the conjugate and degree of dopa conjugation was analyzed by ^1^H-NMR (D_2_O, Bruker 700 MHz; Bruker, Billerica, MA, USA) and UV spectroscopy at 280 nm (Nanodrop 2000; Thermo Scientific, Waltham, MA, USA). ^1^H-NMR chemical shifts are taken at 0.0 ppm with reference to the chemical shift of tetramethylsilane (TMS) added as an internal standard.

### 2.3. Formation and Characterization of Dopa-HA/CaP/pLuc

A plasmid DNA encoding a firefly luciferase gene (pLuc) was used for the formation, characterization, and transfection of dopa-HA/CaP/pLuc nanoparticles. The dopa-HA/CaP/pDNA was formed by dopa-HA coating onto the surface of CaP nanocrystals [32,33]. First, 50 μL of an 18 mM CaCl_2_ solution (pH 9.0) and 50 μL of an 11.5 mM Na_2_HPO_4_ solution (pH 9.0) were mixed for 30 s, and then 15 μL of pDNA (1.5 μg) dissolved in deionized water was added. The dopa-HA (3 μg) was dissolved in 1% acetic acid and reacted with the CaP/pDNA mixture for 20 min. After the reaction, the final pH of the dopa-HA/CaP/pDNA solution became approximately 7.0. The hydrodynamic diameters and surface zeta-potential values of the nanoparticles were investigated by a dynamic light scattering (DLS) device (Zetasizer Nano ZS90 system; Malvern Instruments, Worcestershire, UK) using a laser (He–Ne) wavelength of 633 nm and a scattering angle of 90°. The morphology of the nanoparticles was observed by TEM (JEM-3010; JEOL, Tokyo, Japan).

### 2.4. Cell Culture and Viability Assay

Human mesenchymal stem cells (hMSCs) derived from bone-marrow were purchased from Merck (SCC034, Burlington, MA, USA). hMSCs were seeded in a 96-well plate at a density of 7 × 10^3^ cells/well and maintained in a DMEM medium supplemented with 10% FBS and 1% penicillin/streptomycin at 37 °C in a humidified atmosphere of 5% CO_2_. hMSCs within 7 to 8 passages were used. After 1 day, the cells were incubated for 24 h with various concentrations of dopa-HA conjugates. The cells were then treated with MTT solution (5 mg/mL) at 20 μL/well and further incubated for 2 h. Finally, the medium was replaced with 200 μl of DMSO and incubated for an additional 20 min at 37 °C to dissolve the insoluble formazan crystals. The absorbance was determined at a wavelength of 490 nm using a microplate reader (Multiskan GO; Thermo Scientific, Waltham, MA, USA). The relative cell viability was calculated in comparison to the untreated control cells (100% survival).

### 2.5. In Vitro Transfection and HA Competition Assay

Transfection efficiency was evaluated by measuring the expression of an exogenously transferred reporter gene, pLuc. For the transfection of dopa-HA/CaP/pLuc, hMSCs were seeded in a 12-well plate at a density of 1.5 × 10^5^ cells/well 24 h prior to treatment. The dopa-HA/CaP/pDNA was made from luciferase encoding pDNA (pLuc, 1.5 μg). The cells were treated with nanoparticles in a fresh serum-free medium. After 6 h incubation, the medium was substituted with a fresh medium containing 10% FBS and further incubated for 24 h. Luciferase expression was determined using a luciferase assay system kit and GloMax 20/20 luminometer (Promega, Madison, WI, USA). The expression efficiency of pDNA was normalized to the protein concentration of total cells by BCA assay according to manufacturer’s protocol using the Pierce^TM^ BCA protein assay kit (Thermo Scientific). 

To investigate the HA receptor (CD44)-mediated cellular uptake of dopa-HA/CaP/pLuc in hMSCs, an excess amount (10 mg/mL) of unmodified HA was added to cells 1 h ahead of transfection to prevent interaction between dopa-HA/CaP/pLuc and CD44 receptors. The hMSCs were transfected using dopa-HA/CaP/pLuc with a serum-free medium in the presence of free HA. After transfection, the luciferase expression efficiency of hMSCs was determined as described above. 

### 2.6. Cellular Uptake Studies

EMA-labeled pLuc was used for the formation of dopa-HA/CaP/pLuc because EMA has an emission peak of 625 nm, which is used for florescence-based qualitative and quantitative analysis. The pDNA was labeled with EMA following a previously described procedure [34]. In brief, 20 µL of EMA dissolved in ethanol at a concentration of 5 mg/ml was added to a solution of pDNA dissolved in 200 µg/mL of distilled water. After 30 min incubation on ice, the mixture was exposed to UV light for 20 min. The pLuc was purified by centrifugation and washed with 70% ethanol to remove residual salt. The supernatant was removed and the pLuc was re-suspended in distilled water. The concentration was measured by Nanodrop 2000 (Thermo Scientific).

hMSCs were plated on a glass bottom dish at a density of 3 × 10^5^ cells/well and incubated for 24 h at 37 °C in a humidified atmosphere of 5% CO_2_. The culture medium was then replaced with a fresh serum-free medium containing dopa-HA/CaP/EMApLuc. After 6 h incubation, the cells were washed with phosphate-buffered saline (PBS). The cells were fixed by a 10% buffered formaldehyde solution at 4 °C for 20 min and washed twice with PBS. The nuclei were stained with 4′,6-diamidino-2-phenylindole (DAPI; Thermo Scientific). After staining, the cells were covered with mounting solution to confirm the cellular uptake image using Zeiss LSM 700 laser scanning confocal microscopy (Carl Zeiss Microimaging GmbH, Jena, Germany). For flow cytometry analysis, the transfected cells were prepared as above but trypsinized without fixation. The cellular uptake of EMApDNA was monitored using a Guava easyCyte^TM^ Flow cytometer (Merck Millipore, Burlington, MA, USA) and InCyte software for data acquisition and analysis. 

### 2.7. Construction of pBMP-2

Specific primers were designed from the sequence of bone morphogenic protein 2 (BMP-2) cDNA obtained from GenBank (Accession Number NM_001200.2). To amplify BMP-2 cDNA, PCR was performed with i-pfu DNA polymerase kit (iNtRON Biotechnology Inc., Gyeonggi, Korea) using two oligonucleotide primers (5’-GCGCGCGAATTCATGGTGGCCGGGACCCGC-3’ and 5’-GCGCGCGGATCCCTAGCGACACCCACAACC-3’). Each sample was cycled through 25 thermo-cycles consisting of denaturation for 30 s at 95 °C, annealing for 30 s at 50 °C, and polymerization for 90 s at 72 °C. A final extension for 10 min at 72 °C was performed. The BMP-2 cDNA and mammalian expression vector (flag-tagged-pIRES-CMV) were purified with a MEGA quick-spin^TM^ total fragment DNA purification kit (iNtRON Biotechnology Inc.). Both the BMP-2 cDNA insert and pDNA vector were treated with restriction enzymes (EcoR1 and BamH1) for 2 h at 37 °C. The digested DNAs were purified using the DNA purification kit. The pBMP-2 expression vector was constructed by T4 DNA ligase for 1 h at room temperature. After ligation, the pBMP-2 was transformed into bacterial cells for multiplication. The amplified pBMP-2 was obtained using the plasmid DNA purification kit. The pBMP-2 was used for differentiation experiments. The expression of BMP-2 was confirmed by western blot after transfection of pBMP-2 into hMSCs with dopa-HA/CaP/pBMP-2.

### 2.8. Detection of Osteogenic Differentiation Markers in hMSCs

hMSCs (1.5 × 10^5^ cell/well) were transfected with dopa-HA/CaP/pBMP-2+mir148b. In order to observe the change of expression levels of osteogenic differentiation markers in hMSC, including alkaline phosphatase (ALP), osteocalcin (OC), osteonectin (ON), and osteopontin (OP), total RNA was extracted from the transfected cells using QIAshredder and RNeasy kits (Qiagen Inc., Hilden, Germany) according to the manufacturer’s instructions. The isolated RNA (1 μg) and oligo dT primers were utilized to synthesize single-stranded cDNA using the iScript^TM^ cDNA synthesis kit (Bio-Rad, Hercules, CA, USA). A quantitative real-time PCR was performed using KAPA SYBR fast universal qPCR kit (KAPA Biosystems, Madison, WI, USA) along with 2 μL of cDNA and gene-specific primers at a final volume of 10 μl. The intensity of SYBR green dye was analyzed using CFX Manager^TM^ software. All reactions were carried out in duplicate. The primers and probes of BMP-2, Noggin, ALP, OC, ON, and OP are shown in Table S1.

### 2.9. Histochemical Staining

Osteogenic differentiation of hMSCs was observed 2 weeks after transfection by von Kossa and Alizarin Red staining. For von Kossa staining, after the culture medium was removed, the cells were submerged in 5% silver nitrate solution and incubated under a UV lamp. After 1 h, the cells were treated with 5% sodium thiosulfate, left at room temperature for 3 min, and washed three times with PBS. The cells were treated with the nuclear fast red staining solution and incubated at room temperature for 5 min. The staining images were obtained with a camera. For Alizarin Red staining, and the cells were rinsed three times with PBS (without Ca^+^ and Mg^+^), fixed with 10% paraformaldehyde for 30 min at room temperature, and rinsed with distilled water. The cells were stained with 2% Alizarin Red, incubated at room temperature for 45 min, and washed three times with deionized water and once with PBS before obtaining images.

## 3. Results and Discussion

### 3.1. Formation and Characterization of Dopa-HA/CaP/pLuc

**Figure 1:**
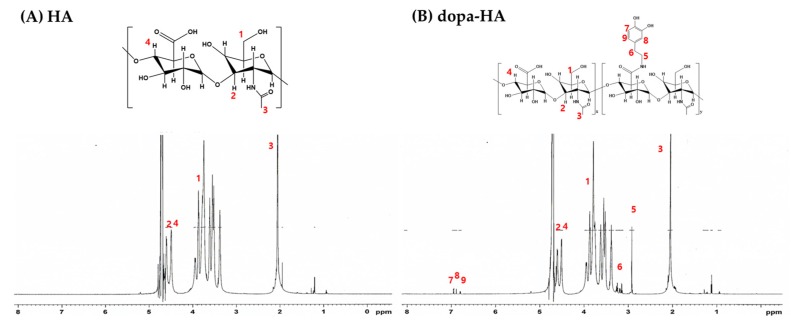
^1^H-NMR spectra of (**A**) HA and (**B**) dopa-HA.

The dopa-HA conjugate was synthesized according to a previous study [33,35]. Briefly, dopamine (dopa) was conjugated to the carboxyl group of HA using EDC/NHS chemistry. The conjugation of dopamine and HA was determined by ^1^H-NMR analysis (Figure 1A,B) and UV spectroscopy (Figure S1). In the ^1^H-NMR spectrum, the glucosidic H peaks were identified at 3.0 to 4.0 ppm, and the peak at 2.03 ppm was associated with hyaluronic acid protons of the -N-COCH_3_ group. The protons of the glucopyranose rings of HA peaked from 4.0 to 5.0 ppm [36,37]. The region near 7.0 ppm confirmed the conjugated dopamine ring. The content of catechol in the dopa-HA conjugate was determined using UV spectroscopy at 280 nm with a standard curve established by distinct concentration of free dopamine. According to the measured absorbance, the degree of dopamine substitution was ca. 5%.

**Scheme 1:**
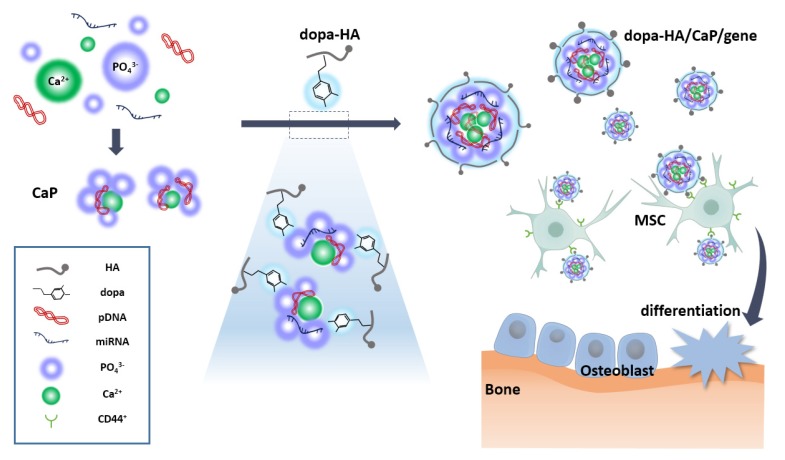
Schematic illustration of dopa-HA/CaP-based gene delivery to mesenchymal stem cells.

**Figure 2:**
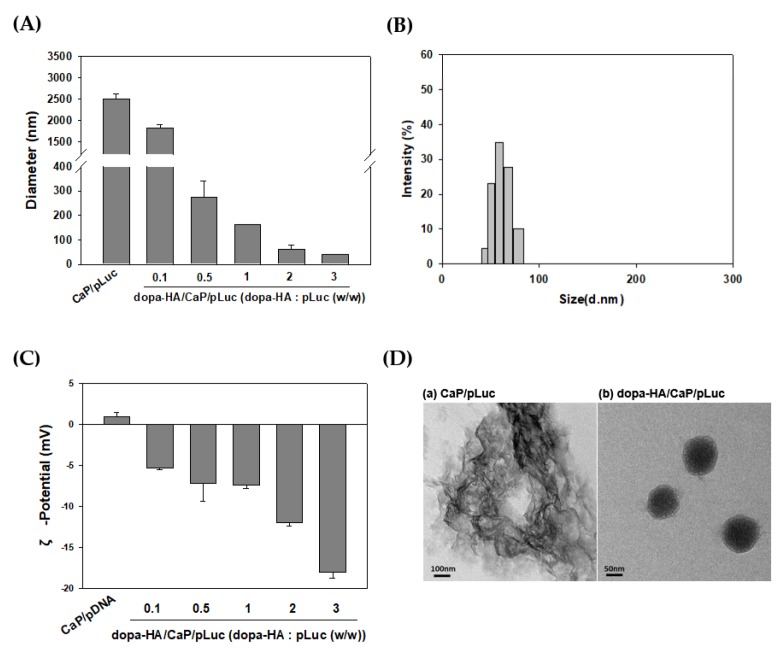
(**A**) Hydrodynamic diameter of dopa-HA/CaP/pLuc at various weight ratios. Values are given as the mean ± SD of triplicates. (**B**) Hydrodynamic diameter of dopa-HA/CaP/pLuc (w/w = 2). (**C**) Surface zeta-potential of dopa-HA/CaP/pLuc at various weight ratios. Values are given as the mean ± SD of triplicates. (**D**) TEM images of (a) CaP/pLuc and (b) dopa-HA/CaP/pLuc (w/w = 2). Size bar = 100 nm (a) and 50 nm (b).

The dopa-HA/CaP/pLuc was prepared as described in Section 2.3. The hydrodynamic diameter and zeta potential of dopa-HA/CaP/pLuc at different weight ratios of pLuc and the dopa-HA conjugate were determined by a dynamic light scattering method. As shown in Figure 2A, the hydrodynamic diameter of dopa-HA/CaP/pLuc gradually decreased with increasing weight ratio of the complexes. In particular, dopa-HA/CaP/pLuc at the weight ratio (dopa-HA/pLuc, w/w) of 2 exhibited 71 ± 7 (PDI: 0.43 ± 0.09), as determined by a dynamic light scattering method (Figure 2B). The surface zeta-potential of dopa-HA/CaP/pLuc presented a negative charge due to the carboxyl group of the HA backbone (Figure 2C). TEM images showed that dopa-HA/CaP/pLuc were compact and spherical, while CaP/pLuc formed large aggregates after 25 min incubation (Figure 2D). These results indicate that the catechol moieties of the dopa-HA conjugate serve as a tight binding anchor on the surface of CaP crystals to retain the stability of the nanoparticles. The catechol group has been used for the chemical functionalization of material surface as well as for the nucleation of natural inorganic crystals. The HA on the surface of dopa-HA/CaP/pLuc could restrict undesirable aggregation and excessive crystal growth and specifically bind to CD44 of hMSCs, leading to efficient internalization of the nanoparticles into cells (Scheme 1).

### 3.2. In Vitro Transfection of Dopa-HA/CaP/pLuc

**Figure 3:**
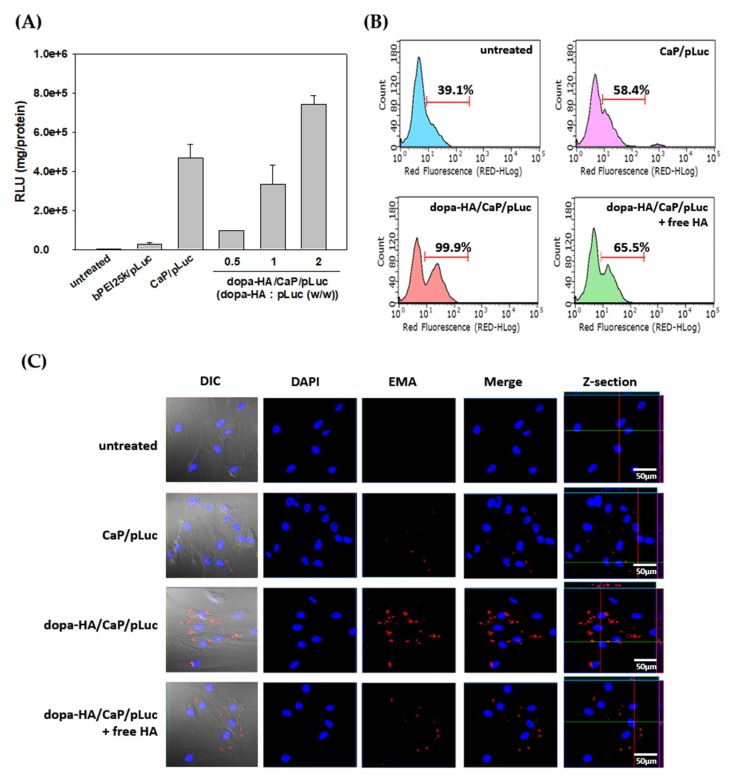
(**A**) Transfection efficiency of dopa-HA/CaP/pLuc (w/w = 2) in human mesenchymal stem cells (hMSCs). The bPEI 25 kDa (bPEI25k/pLUC, w/w = 1) and untreated cells were used as controls. Flow cytometry (**B**) and confocal microscopy (**C**) results of the cellular uptake of CaP/pLuc and dopa–HA/CaP/pLuc (w/w = 2) in the absence or presence of free HA.

HA and dopa-HA showed no cytotoxicity in hMSCs (Figure S2). The transfection efficiency of dopa-HA/CaP/pLuc was evaluated by measuring the expression of a reporter gene after transfection of pLuc to hMSCs. As shown in Figure 3A, luciferase expression by dopa-HA/CaP/pLuc increased as the weight ratio (dopa-HA/pLuc, w/w) increased. The dopa-HA/CaP/pLuc exhibited higher expression than CaP/pLuc at the weight ratio of 2, suggesting that the addition of dopa-HA stabilized the surface of the nanoparticle, leading to improved transfection efficiency. To observe the intracellular delivery of the dopa-HA/CaP/pLuc, pLuc was labeled with ethidium monoazide (EMA) as a fluorescent probe, and the cells were observed by flow cytometry and confocal microscopy. The results demonstrated that dopa-HA/CaP/pLuc can mediate higher cellular uptake than CaP/pLuc, which suggests that the enhanced transfection efficiency of dopa-HA/CaP/pLuc was due to increased cellular delivery of the reporter pDNA. The specific interactions between HA and CD44 were further investigated by adding free HA to the transfection medium. In the presence of free HA, the number of cells transfected by dopa-HA/CaP/pLuc decreased from 99.9% to 65.5% in the arbitrarily gated region (Figure 3B). The confocal images supported the flow cytometry results, showing that the fluorescence was localized inside the cells (Figure 3C). Pretreatment of free HA reduced the internalization of dopa-HA/CaP/pLuc into hMSCs, consistent with the flow cytometry analysis (Figure S3). The enhanced cellular delivery led to increased expression of the reporter gene in hMSCs (Figure 3C and Figure S4). These results suggest that the enhanced transfection of dopa-HA/CaP/pLuc is closely related to CD44 receptor-mediated endocytosis. 

### 3.3. Osteogenic Differentiation in hMSCs

**Figure 4:**
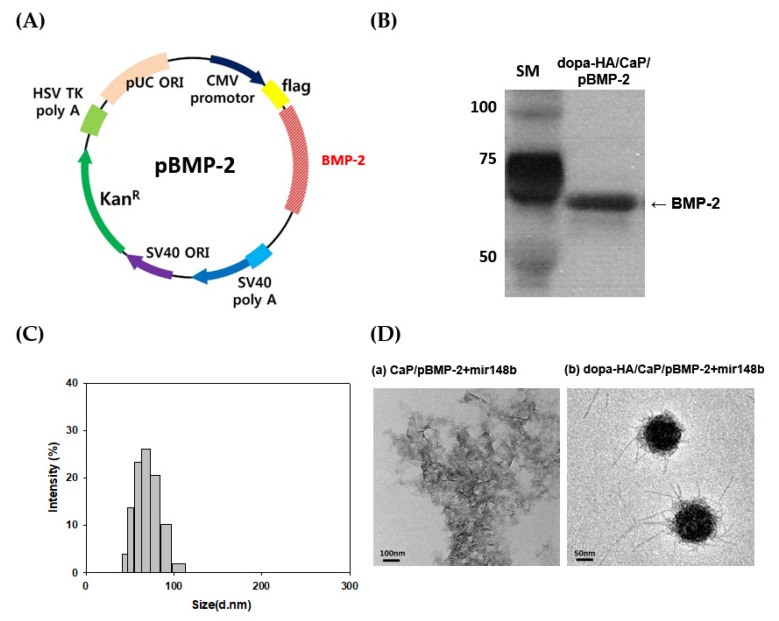
(**A**) Plasmid map of BMP-2 pDNA and (**B**) western blot analysis of BMP-2 protein using dopa-HA/CaP/pBMP-2. (**C**) Hydrodynamic diameter of dopa-HA/CaP/pBMP-2+mir148b. (**D**) TEM images of CaP/ pBMP-2+mir148b (a) and dopa-HA/CaP/pBMP-2+mir148b (b) (w/w = 2).

A pDNA encoding BMP-2 (pBMP-2) was constructed using a recombinant DNA technique (Figure 4A). The expression of the BMP-2 protein in hMSCs after the transfection of dopa-HA/CaP/pBMP-2 was confirmed by western blot analysis (Figure 4B). To improve osteogenic differentiation of hMSCs, we attempted to deliver two nucleic acids, pBMP-2 and mir148b. BMP-2 protein expressed from pBMP-2 can activate the upstream signals for osteogenic differentiation of hMSCs, and, at the same time, the simultaneously delivered mir148b can down-regulate the antagonistic extracellular modulator, Noggin, in the cells. The resulting nanoparticle containing pBMP-2 and mir148b (dopa-HA/CaP/pBMP-2+mir148b) had a diameter of 78 ± 9 nm (PDI: 0.43 ± 0.14) with a spherical morphology (Figure 4C). The hydrodynamic diameter of dopa-HA/CaP/pBMP-2+mir148b were not significantly changed compared to the nanoparticle containing pDNA alone (*p* = 0.295). The morphology of dopa-HA/CaP/pBMP-2+mir148b was also spherical, as shown in Figure 4D. This may be because both pDNA and miRNA, having similar physicochemical properties, were physically entrapped in the inorganic matrix of CaP. 

**Figure 5:**
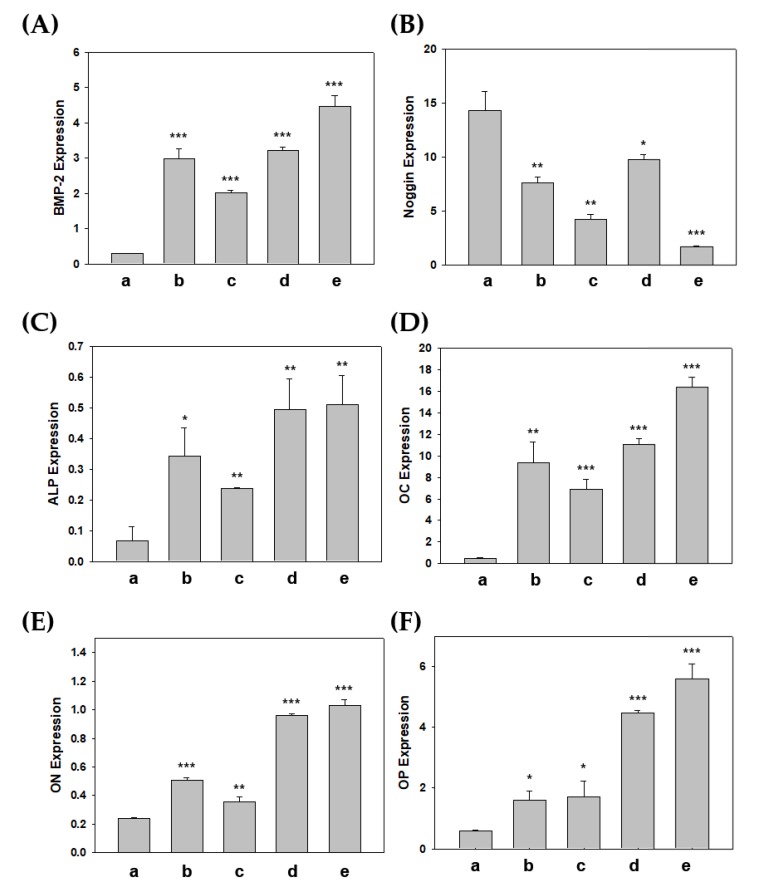
Expression profiles of (**A**) BMP-2 and (**B**) Noggin in hMSCs after transfection. Expression levels of osteogenic differentiation markers, including (**C**) alkaline phosphatase (ALP), (**D**) osteocalcin (OC), (**E**) osteonectin (ON), and (**F**) osteopontin (OP), were analyzed by quantitative RT-PCR. Experiments were carried out in duplicate and expressed as a mean ± SD. a. untreated; b. CaP/pBMP-2+mir148b; c. dopa-HA/CaP/mir148b; d. dopa-HA/CaP/pBMP-2; e. dopa-HA/CaP/pBMP+mir148b. Statistical difference between a test group and untreated control (a) was tested using Student’s *t*-test. * *p* < 0.05, ** *p* < 0.01, *** *p* < 0.001.

The cellular delivery of dopa-HA/CaP/pBMP-2+mir148b led to expression of the BMP-2 protein and suppression of the Noggin gene in hMSCs at the same time (Figure 5A,B). The differentiation of hMSCs by dopa-HA/CaP/pBMP-2+mir148b was evaluated by observing the expression profiles of a set of marker genes closely involved with osteogenic differentiation, including alkaline phosphatase (ALP), osteocalcin (OC), osteonectin (ON), and osteopontin (OP). The gene expression levels were determined by quantitative RT-PCR. These results demonstrated that the expression of osteogenic BMP-2 and reduction of Noggin that antagonizes BMP activity by the co-delivery of pBMP-2 and mir148b using dopa-HA/CaP can induce the osteogenic differentiation of hMSCs. 

**Figure 6:**
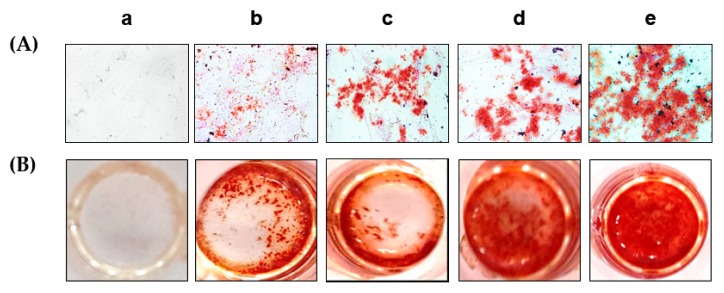
Induction of osteogenic differentiation of hMSCs. (**A**) von Kossa staining image of hMSCs after transfection with the indicated formulation. (**B**) Calcification of the cells detected by Alizarin Red S staining. The assays were carried out two weeks after the transfection. (a) untreated; (b) CaP/pBMP-2+mir148b; (c) dopa-HA/CaP/mir148b; (d) dopa-HA/CaP/pBMP-2; (e) dopa-HA/CaP/pBMP+mir148b.

The osteogenic differentiation of hMSCs was confirmed by observing the intracellular calcium deposition using von Kossa and Alizarin Red staining (Figure 6). Consistent with the expression profiles of the osteogenic marker genes, the transfection of dopa-HA/CaP/pBMP+mir148b resulted in significantly higher mineralization of the cells than other control groups, suggesting that concurrent delivery of the differentiation modulators, BMP-2 and mir148b, would be one desirable way to increase the differentiation efficiency of hMSCs. 

## 4. Conclusions

In this study, inorganic CaP-based nanoparticles stabilized by a catechol-derivatized organic polymer (dopa-HA) were designed for gene delivery to hMSCs. Owing to their ability to entrap any type of nucleic acid, the surface-stabilized CaP nanoparticles enabled the efficient cellular delivery of a plasmid DNA (pBMP-2) and a micro RNA (mir148b) to hMSCs via CD44-specific receptor-mediated endocytosis. The osteogenic differentiation of hMSCs was significantly improved by the expression of BMP-2 and down-regulation of Noggin, an antagonist for BMP-2-related upstream signal transduction. Considering its biocompatibility, simplicity of preparation, and enhanced gene transfection to hMSCs via CD44 receptor-mediated endocytosis, this dopa-HA/CaP-based gene delivery system is an attractive candidate for transferring multiple genes to hMSCs for improved differentiation.

## Supplementary Materials

The following are available online at https://www.mdpi.com/2073-4360/11/5/798/s1, Figure S1: UV spectroscopy of HA and dopa-HA. Figure S2: Cell viability of hMSCs after treatment of HA and dopa-HA at different concentrations. Figure S3: Gene transfection efficiency of dopa–HA/CaP/pLUC (w/w = 2) in hMSCs either in the absence and presence of free HA. Figure S4: Comparison of the cellular uptake of CaP/pLuc and dopa–HA/CaP/pLuc (w/w = 2) in the absence and presence of free HA. Values are obtained from the confocal images in Figure 3C by measuring the red fluorescence signal intensity of the pixels using Image J software. Table S1: Description of the primers for quantitative RT-PCR.
