# Supplementary material for: Enhanced Transfection of Human Mesenchymal Stem Cells Using a Hyaluronic Acid/Calcium Phosphate Hybrid Gene Delivery System
Source: Polymers (Basel). 2019 May 4;11(5):798. doi: 10.3390/polym11050798 (PMC6571843; doi:10.3390/polym11050798)
Supplement: Supplementary file 1 [file polymers-11-00798-s001.pdf]

polymers

Supplementary information

Enhanced Transfection of Human Mesenchymal Stem Cells Using Hyaluronic Acid/Calcium Phosphate Hybrid Gene Delivery System

Jung Eun Lee, Yue Yin, Su Yeon Lim, E Seul Kim, Jaeback Jung, Dahwun Kim, Ji Won Park, Min Sang Lee* and Ji Hoon Jeong *

School of Pharmacy, Sungkyunkwan University, Suwon 16419, Korea

* Correspondence: lminsa@skku.edu (M.S.L.); jhjeong@skku.edu (J.H.J.); Tel.: +82-31-290-7783 (J.H.J.)

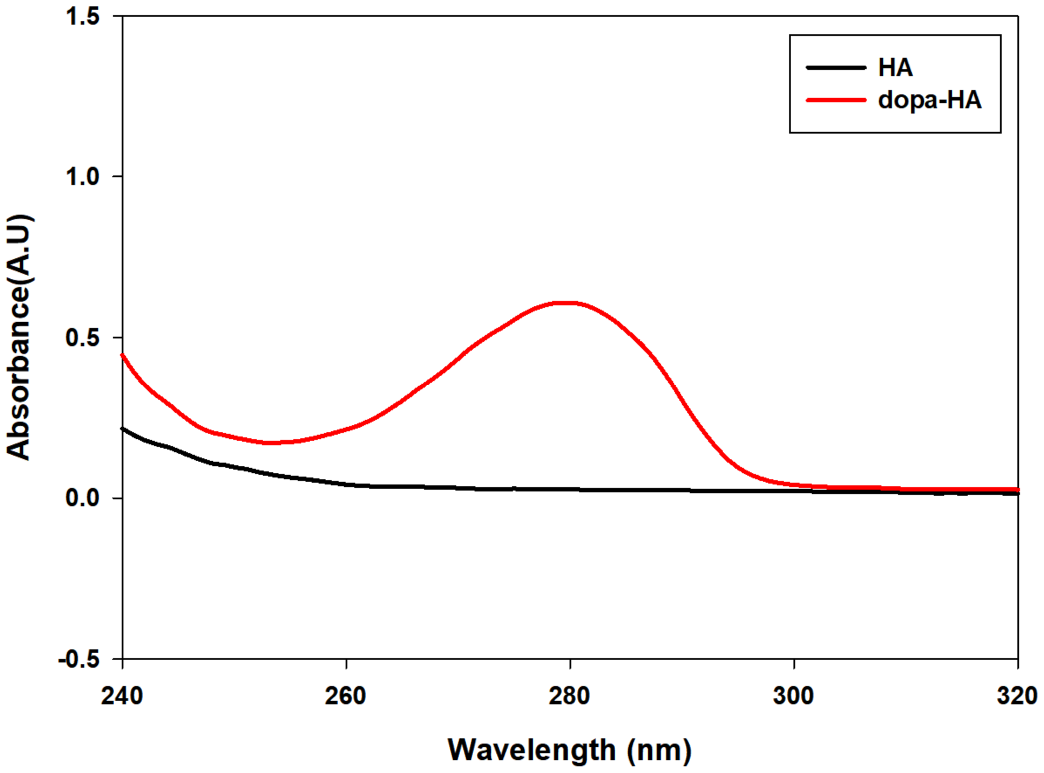

Figure 1. UV spectroscopy of HA and dopa-HA.

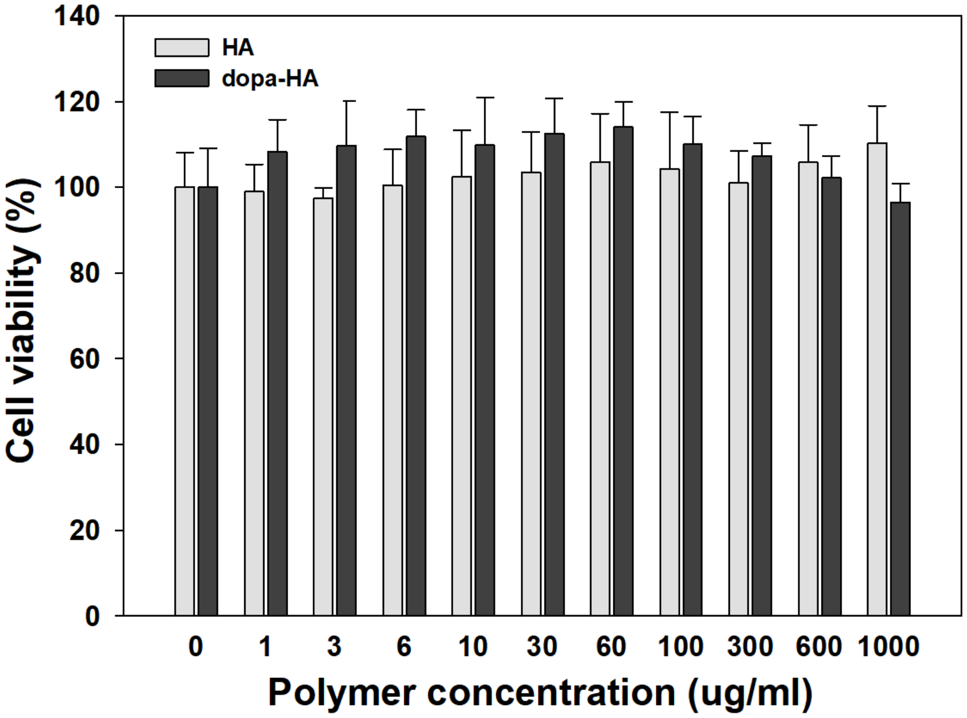

Figure 2. Cell viability of hMSCs after treatment of HA and dopa-HA at different concentrations.

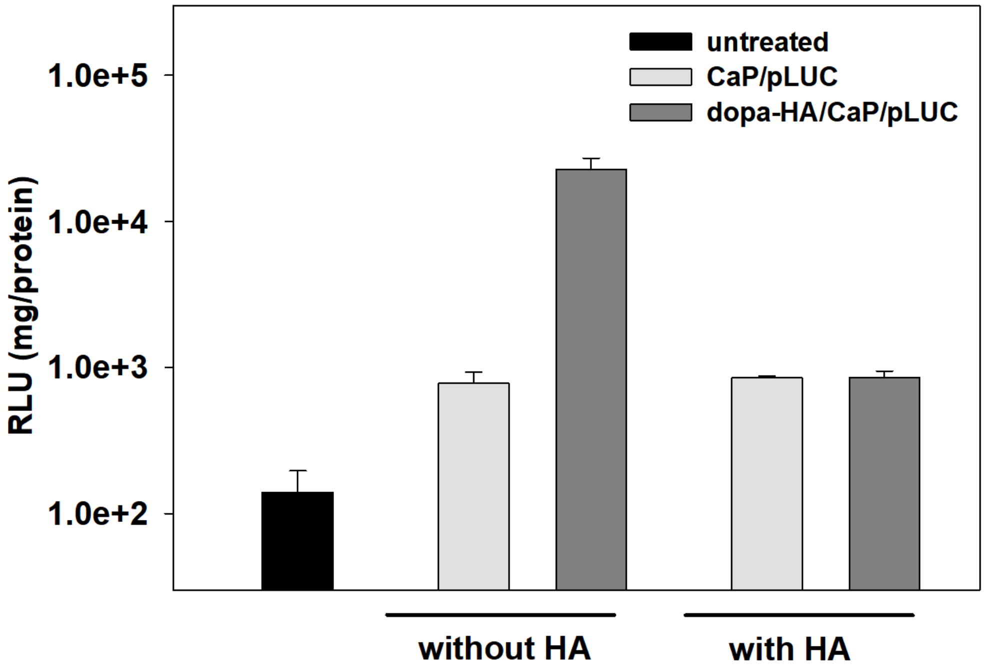

Figure S3. Gene transfection efficiency of dopa–HA/CaP/pLUC (w/w=2) in hMSCs either in the absence and presence of free HA. Values are given as the mean ± SD of triplicates.

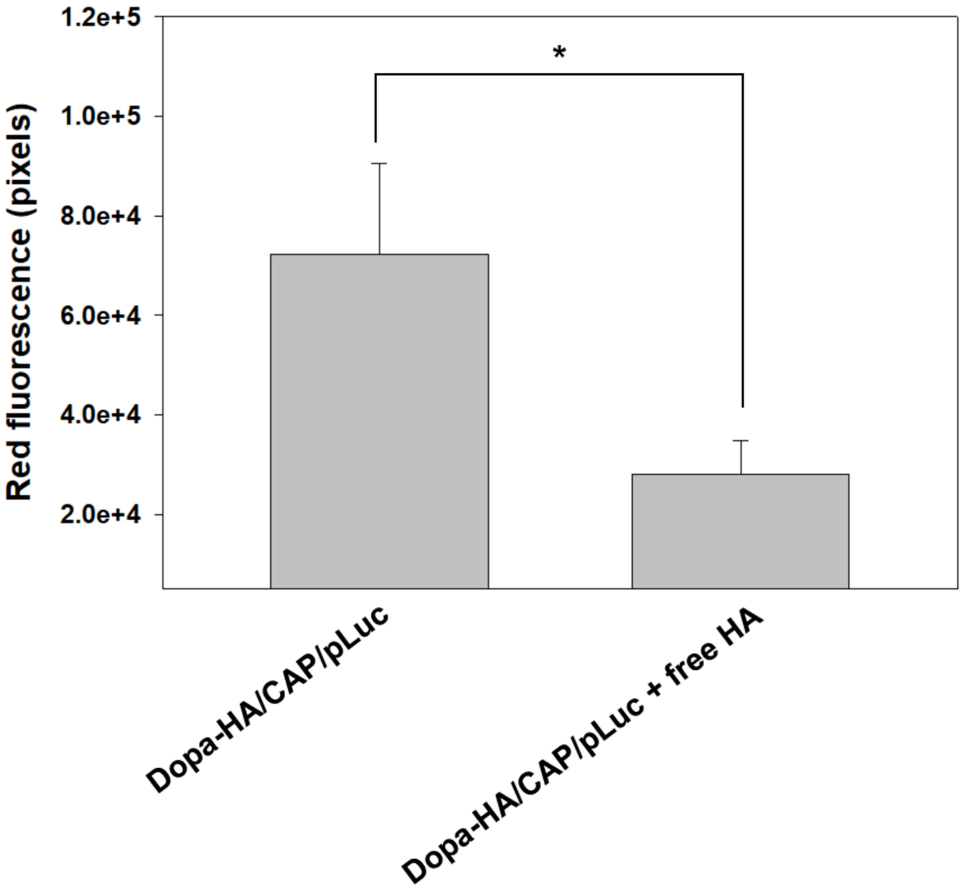

Figure S4. Comparison of the cellular uptake of CaP/pLuc and dopa–HA/CaP/pLuc (w/w= 2) in the absence and presence of free HA. Values are obtained from the confocal images in Figure 3C by measuring the red fluorescence signal intensity of the pixels using Image J software. * $p < 0.05$.

Table 1. Description of the primers for quantitative RT-PCR.

Lineage	Gene	Oligonucleotide primers
**Bone**	BMP2	5’-AGTTGCGGCTGCTCAGCATGTT
		5’-ACATGTCTCTTGGAGACACCT
	Noggin	5’-GAGGAAGTTACAGATGTGGCT
		5’-CACTCGGAAATGATGGGGTAC
	ALP	5’-ACATTCCCACGTCTTCACATTT
	(Alkaline phosphatase)	5’-AGACATTCTCTCGTTCACCGCC
	OC	5’-ACCTTTGCTGGACTCTGCAC
	(osteocalcin)	5’-TGAGAGCCCTCACACTCCTC
	ON	5’-TTCCCTGTACACTGGCAGTTC
	(osteonectin)	5’-AATGCTCCATGGGGATGA
	OP	5’-TTTCGCAGACCTGACATCC
	(osteopontin)	5’-GGCTGTCCCAATCAGAAGG
	β-actin	5’-CCAACCGCGAGAAGATGA
		5’-CCAGAGGCGTACAGGGATAG
